# Adolescent Suicidal Behavior and Substance Use: Developmental Mechanisms

**DOI:** 10.4137/sart.s1044

**Published:** 2008-10-31

**Authors:** Michael A Dawes, Charles W Mathias, Dawn M Richard, Nathalie Hill-Kapturczak, Donald M Dougherty

**Affiliations:** All of the authors are in the Department of Psychiatry, Division of Alcohol and Drug Addiction, Neurobehavioral Research Laboratory and Clinic, 7703 Floyd Curl Drive, MC 7792, San Antonio, TX 78229-3900, United States of America.

**Keywords:** adolescence, development, suicide, substance abuse, behavior, risk and protective factors

## Abstract

Adolescent suicidal behaviors and substance use are disturbingly common. Research suggests overlap of some of the etiological mechanisms for both adolescent suicidal behavior and substance use, yet clear understanding of the complex relations between these behaviors and their causal underpinnings is lacking. A growing body of evidence and a diathesis model (Mann et al. 1999; Mann, 2003) highlight the importance of impulse control as a proximal risk factor for adolescent suicidal and substance use behaviors. This literature review extends current theory on the relationships between adolescent suicidal behavior and substance use by: (1) examining how, when, and to what extent adolescent development is affected by poor impulse control, stressful life events, substance use behavior, and biological factors; (2) presenting proposed causal mechanisms by which these risk factors interact to increase risk for suicidal behaviors and substance use; and (3) proposing specific new hypotheses to extend the diathesis model to adolescents at risk for suicide and substance use. More specifically, new hypotheses are presented that predict bidirectional relationships between stressful life events and genetic markers of 5-HT dysregulation; substance use behavior and impulsivity; and substance use behavior and suicide attempts. The importance of distinguishing between different developmental trajectories of suicidal and substance use behaviors, and the effects of specific risk and protective mechanisms are discussed. Use of new statistical approaches that provide for the comparison of latent growth curves and latent class models is recommended to identify differences in developmental trajectories of suicidal behavior and substance use. Knowledge gained from these prospective longitudinal methods should lead to greater understanding on the timing, duration, and extent to which specific risk and protective factors influence the outcomes of suicidal behavior and substance use. In turn, findings from these studies should inform researchers who conduct future treatment and prevention studies.

## Epidemiological Findings of Adolescent Suicidal Behavior and Substance Use

Adolescent suicidal behaviors are widespread and produce a significant burden on healthcare systems. In the United States, suicide is the 4th most common cause of death among 10–14 year olds, and the 3rd most common cause of death among 15–24 year olds (Anderson and Smith, 2003). Suicide attempts are the primary reason for referral to child and adolescent psychiatric emergency services (Peterson et al. 1996). The incidence rates of suicide attempts among older adolescents range from 7% to 9% (CDC, 2004). Prospective findings show that: (1) adolescents who attempt suicide are at risk of future non-lethal suicide attempts, and the risk increases with multiple suicide attempts (Goldston et al. 1999; Wingate et al. 2004); (2) adolescents who die by suicide have histories of suicidal ideation (Beck et al. 1999) and non-lethal suicide attempts (Shaffer et al. 1996); and (3) the period from 6 to 12 months following discharge from psychiatric hospitalization is marked by a heightened risk for suicide attempts (Goldston et al. 1999).

**Figure 1:**
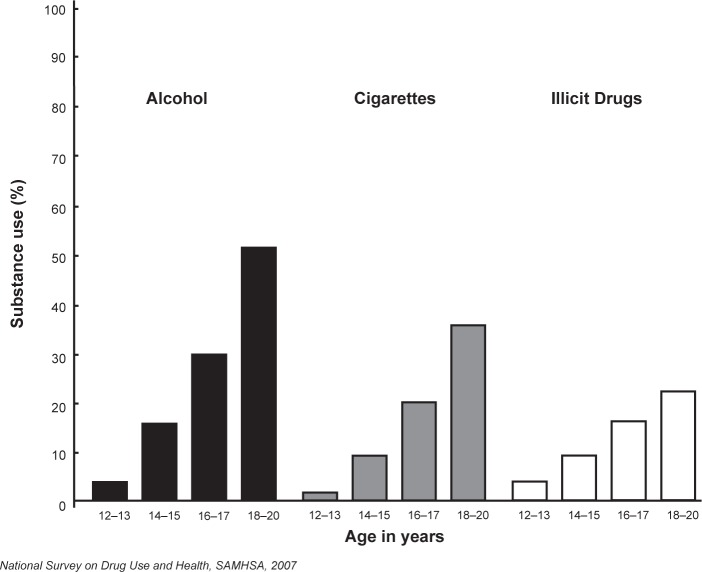
Adolescent substance use reported in the past month.

In addition to suicidal behavior, adolescence is also the developmental period when drug experimentation frequently occurs, often progressing to substance dependence and substance-related problems. Estimates from a recent community-based survey of adolescents (12–17 year olds) indicated that 33% (more than 8 million) reported alcohol use, 20% (nearly 5 million) reported illicit substance use, and 17% (nearly 4 million) reported cigarette use during the past year (National Survey on Drug Use and Health, SAMHSA, OAS, 2007). Furthermore, self-reported use of alcohol, cigarettes, or other drug use increases systematically during adolescence (see Fig. 1). Risk for substance dependence is elevated for: alcohol between 15 and 35 years old (peaking at about age 20); marijuana between 15 and 25 years old (peaking at age 17); and cocaine between 15 and 30 (Wagner and Anthony, 2002). Early drug experimentation predicts later development of regular substance use, substance-use disorders, and substance-related problems (Anthony and Petronis, 1995; de Wit et al. 2000; Fergusson et al. 1996). Furthermore, substance use during adolescence may produce long-term negative effects on neurocognitive and behavior functioning (Ehrenreich et al. 1999; Schneider and Koch, 2003; Volkow, 2005). For example, adolescents who initiate and progress to regular marijuana and other substance use undergo changes in behavioral, affective, and cognitive processes characterized as an underdeveloped regulation of aggression, sensation seeking, and impulsivity (Hawkins et al. 1992; McGue et al. 2001a, 2001b; Volkow, 2005).

Cross-sectional and retrospective studies of both adolescents and adults have typically reported an association between suicidal behaviors and substance use. In the U.S., the 20-year period following the rapid increase in drug abuse (beginning in the 1960’s) also included a 300% increase in adolescent suicidal behaviors (Goldsmith et al. 2002). Adolescent substance users have more than a 2.5-fold increase in risk for suicidal behaviors compared to non-drug using adolescents (SAMHSA, 2002). Among adults admitted to drug-abuse treatment centers, 28% reported histories of suicidal ideation and 21% reported histories of suicide attempts (Wines et al. 2004). During the 2 years following discharge from drug-abuse treatment, significant percentages of these adults continued to report suicidal ideation and reoccurring suicide attempts (19% and 7%, respectively; Wines et al. 2004). Finally, the progression of substance use severity has been related to corresponding increases in the severity of suicidal behaviors (Garrison et al. 1993).

## Developmental Features of Adolescence

Adolescence is a critical developmental period of heightened risk for both suicide and substance use that includes multiple interactions between the brain, behavior, and social environment (Dahl, 2004) that affect maturation (Chambers et al. 2003; Dahl, 2004; Pratt, 2002; Steinberg, 2005). These interactions can occur with specific risk factors, to increase the likelihood of suicide and substance use. In this section, we highlight developmental features of adolescence, then, in the next section, discuss particular risk factors related to adolescent suicidal behavior and substance use.

Adolescence begins with the onset of puberty and ends with the establishment of adult roles and responsibilities (Chambers et al. 2003; Dahl, 2004; Paus, 2005; Steinberg, 2005). With healthy development, adolescents move relatively smoothly through multiple domains of maturation, including physical growth and pubertal maturation, perceptual-motor, visual, cognitive, auditory, language, kinesthetic, and psychosocial integration and adaptation (Dahl, 2004; Pratt, 2002). During adolescence, motivated behavior progresses from simple play to a greater inclination to seek experiences that create high-intensity feelings and emotions, excitement, and adult-like experiences (Chambers et al. 2003; Dahl, 2004). As they approach adulthood, healthy adolescents become increasingly more capable of controlling impulsive behaviors, in part, because of maturation in the prefrontal cortex and other brain circuits that mediate impulse control (Chambers et al. 2003; Dahl, 2004).

Despite the fact that impulse control increases during adolescence with normal development, affective, cognitive, and behavior processes are still immature. Immaturity in these processes may lead to a typical adolescent’s tendency to act impulsively, be emotionally reactive, and ignore the negative consequences of their behavior. Impulsive, emotional reactivity without consideration of potential consequences increases the likelihood of risk taking and initiation of substance use (Chambers et al. 2003; Clark et al. 2005; Dahl, 2004; Spear, 2000; Volkow, 2005; Volkow and Li, 2005). Adolescents with severe impulse control problems may have earlier onset of substance use and worse clinical prognoses. For example, among 11 year olds, impulsive behavioral disorders (e.g. Oppositional Defiant Disorder, Conduct Disorder, and ADHD) are predictive of earlier initiation of alcohol use (McGue et al. 2001). Similarly, boys with impulse control problems have a higher risk for development of a substance use disorder, and impulsivity appears to impart this risk by leading to an earlier age of first drink of alcohol (von Diemen et al. 2008). Furthermore, additional factors may be involved in the development of more severe outcomes, such as mood disorders and other psychopathology that often occurs with suicidal behavior (Bridge et al. 2006).

Because of the frequent co-occurrence of suicidal behavior and substance abuse, there is a need to better understand the relationships between these behaviors (Erinoff et al. 2004), as well as the risk factors for these behaviors. Previous studies of the relationship between suicidal and substance use behaviors have focused on the degree to which drug abuse is associated with increased risk for suicidal behavior (Borges et al. 2001; Hawton et al. 1993; Pages et al. 1997; Rivara et al. 1997), and the relationship of substance use behaviors to both suicidal ideation (Levy and Deykin, 1989; Pages et al. 1997) and suicide attempts among adolescents and adults (Beautrais et al. 1997; Levy and Deykin, 1989). However, little is known about the risk factors that are associated with these behaviors. Some important gaps in the suicide literature are an understanding of when and how different risk and protective factors interact to influence the probability for suicidal behaviors and substance use. In the following sections, we first review specific risk factors of suicidal behavior and drug abuse, which includes impulse control and impulsive aggression, stressful life events, and prior substance use behavior, as well as serotonin dysregulation and other biological factors. Second, we present approaches to studying suicidality and substance use, the developmental and conceptual issues to consider, and the methods to conduct innovative longitudinal studies of these behaviors during adolescence. Third, we present a model of suicide (e.g. the stress-diathesis model of suicidal behavior; Mann et al. 1999; Mann, 2003), review features unique to adolescent development, and suggest extensions of the model that may help to explain how, when, and to what extent specific factors increase the risk for adolescent suicidal behavior and substance use.

## Risk Factors of Adolescent Suicidal Behavior and Substance Use

Poor impulse control, defined as “a predisposition toward rapid, unplanned reactions to internal or external stimuli without regard to the negative consequences of these reactions to the impulsive individual or to others” (Moeller et al. 2001; p. 1784) has been described as a core behavioral feature of substance use disorders (Moeller et al. 2001), and is often cited as a common behavioral characteristic among suicidal individuals (e.g. Dougherty et al. 2004b, 2004c; Horesh, 2001; Mann, 1999). Recently, attention has been focused on the interactive effects of substance abuse and poor impulse control in determining suicidal behaviors (Dalton et al. 2005; Dougherty et al. 2004b; Putnins, 1995). Evidence suggests that the combination of poor impulse control, stressful life events, suicidal behavior and substance use may interact to amplify the likelihood of negative consequences that occur with these behaviors (Bridge et al. 2006; Dalton et al. 2005; Putnins, 1995). In this section, we first describe key risk factors for adolescent suicidal behavior and substance use and introduce the concept that developmental-transactional features of adolescence includes interaction with the following risk factors, to increase the risk of suicidal behavior and substance use.

### Poor impulse control

Poor impulse control is a key risk factor that often underlies suicidal and substance use behaviors (Apter et al. 1995; Beautrais et al. 1999; Brent et al. 1988; Dougherty et al. 2004b, 2004c). Poor impulse control is an important feature of both externalizing behaviors (e.g. Conduct Disorder; Daderman, 1999; Dougherty et al. 2003a, 2003b; Gorenstein and Newman, 1980; Klein et al. 1997; Milich and Kramer, 1984; Tranah et al. 1998) and substance use (Dougherty et al. 2004b; Moeller et al. 2002b). Poor impulse control has been linked to the phenomenology, neurobiology, and familial transmission of suicidal behavior (Brent et al. 1996, 2002, 2003, Mann, 1998; Mann et al. 1999), and to adolescent suicidal behavior (Kashden et al. 1993; Kinsbury et al. 1999), even after controlling for hopelessness, neuroticism, external locus of control, and extroversion (Beautrais et al. 1999). Among adolescents, one type of poor impulse control, impulsive aggression (the tendency to react to frustration and/or provocation with aggression), is often prominent in disruptive behavior disorders, and has been shown to predict suicidal behavior (Apter et al. 1995; Beautrais et al. 1999), independent of depression (Apter et al. 1988; Brent et al. 1988, 1993; Cohen-Sandler et al. 1982; Kovacs et al. 1993; Pfeffer et al. 1983).

### Stressful life events

Recent studies have shown that in adolescents and young adults, there are high rates of traumatic (e.g. life threatening) and other, often interpersonal, stressful life events (Hatch and Dohrenwend, 2007). In adolescents and young adults, these stressful life events are more likely to include physical and sexual assaults (Hatch and Dohrenwend, 2007).

Stressful life events, both traumatic and interpersonal, have been shown to contribute to suicide risk in adolescents. Interpersonal stressors are among the most important risk factors for adolescent suicidal behavior, especially when these interpersonal stressors occur during childhood; examples include parental separation, social isolation; poor family communication, family dysfunction; relationship break-ups, conflicts with peers or parents; victimization by peers; low social support, as well as relationship strain due to parental substance abuse and other parental psychopathology (Davies and Cunningham, 1999; Gould et al. 1996; Hawton et al. 1996; Lewinsohn et al. 1994; Wagner, 1997; Zalsman and Mann, 2005). Childhood physical and sexual abuse also increase the risk of adolescent suicidal behavior (Brent et al. 1994; Deykin and Buka, 1994; de Wilde et al. 1992; Horesh et al. 2003; Kendall-Tackett et al. 1993). Academic failure and disciplinary actions contribute to suicide risk among adolescents, including failing a grade, suspension from school, and legal sanctions (Gould et al. 1996; Runeson and Beskow, 1991). Collectively, these kinds of stressful life events are important risk factors for adolescent suicidal behavior.

Stressful life events also are risk factors for the onset and escalation of substance use (Novins and Mitchell, 1998; Petraitis et al. 1995; van den Bree and Pickworth, 2005; Wills et al. 1996; Windle and Wiesner, 2004). Important risk factors for substance abuse include poor family environment (e.g. poor care by family, single parent family, dysfunctional parent-child relationship, family conflict; Ellickson et al. 2004b; Flory et al. 2004), traumatic events (e.g. death of parent or sibling, divorce or serious illness of parent, victim of a crime; Hoffmann, 1995; Nation and Heflinger, 2006; von Sydow et al. 2002; Windle and Wiesner, 2004), and interpersonal as well as intrapersonal events (e.g. accidents or illness, breaking up with a friend, relocation; Brown, 1989; Novins and Mitchell, 1998; Windle and Wiesner, 2004). Thus, stressful life experiences (both traumatic and interpersonal) often cause mental or physical distress, and are thought to increase the likelihood of adolescent substance use involvement and suicidal behaviors.

### Substance use behavior

Adolescent substance use may increase the risk for suicidal behavior due to both acute and long-term effects. Acute pharmacological effects of drug intoxication may impair judgment, lower inhibitions, worsen impulse control, and affect specific neurotransmitter systems (Mann et al. 2003), to increase the likelihood of attempting suicide (Bridge et al. 2006; Schuckit and Schuckit, 1989). Chronic substance use may cause negative effects on neurocognitive and behavioral control (Ehrenreich et al. 1999; Schneider and Koch, 2003; Volkow, 2005). Adolescents who initiate and progress to regular substance use often undergo changes in behavioral, affective, and cognitive processes characterized as an underdeveloped regulation of aggression, sensation seeking, and impulsivity (Clark et al. 2005; Hawkins et al. 1992; McGue et al. 2001a, 2001b). Substance use can also trigger suicidal behavior by contributing to developmental failures, such as school difficulties or expulsion, or problematic interaction with peers (Conner and Goldston, in press).

Exposure to substance use can also be a risk factor for increases in drug involvement. Family influences that increase risk for the initiation of substance use during adolescence include parents or older siblings who use drugs (Ellickson et al. 2004b) and parents or other first-degree biological relatives with histories of substance use disorders (Brook et al. 1999; Clark et al. 2005; Hawkins et al. 1992; Hill et al. 2000; Höfler et al. 1999; Morojele and Brook, 2001; Newcomb and Felix-Ortiz, 1992; Novins and Mitchell, 1998; Petraitis et al. 1995; Tarter et al. 2003; von Sydow et al. 2002; Wills et al. 1996). And peer influences, including peer approval of alcohol or other substance use (Ellickson et al. 2004b; Kosterman et al. 2000; Novins and Mitchell, 1998), and offers of marijuana (Novins and Mitchell, 1998; von Sydow et al. 2002), predict the initiation (Hawkins et al. 1992) and escalation (Morojele and Brook, 2001) of substance use. Finally, personal experience using alcohol and tobacco predicts the initiation and escalation of marijuana and other illicit substance use (Bentler et al. 2002; Boyle et al. 1992; Clark et al. 1998, 2005; Ellickson et al. 2004b; Novins and Mitchell, 1998; van den Bree and Pickworth, 2005).

### Serotonin dysregulation and related biological factors

Serotonin (5-HT) dysregulation may be associated with both suicidal behaviors (Mann et al.1999; Mann, 2002) and substance abuse (e.g. Javors et al. 2005; Morgan, 2000). Alterations in 5-HT function (LeMarquand et al. 1994a, 1994b; Mann, 1998; Owens and Nemeroff, 1994) and other systems involved in cell signaling and signal modulation (Pandey et al. 1997, 2002, 2004) have been linked to suicide and suicidal behavior. Among adults, Malone and colleagues (1996) showed that alterations in central 5-HT function were especially pronounced in suicide attempters who were younger than age 30. In post-mortem studies of adolescents, Pandey and colleagues (1997, 2002, 2004) showed that, compared to deceased controls, adolescent suicide completers had increased 5-HT_2A_ receptor mRNA and protein expression in the prefrontal cortex and hippocampus. Higher levels of 5-HT_2A_ receptors may be one of the neurobiological abnormalities associated with adolescent suicidal behavior. Other biological factors related to completed suicide are decreased protein kinase A and C, down-regulated CREB, and increased activity of brain-derived neurotrophic factor (BDNF) in the prefrontal cortex and hippocampus (Pandey et al. 2004). These latter findings suggest that both the serotonin system, and cell signaling and signal modulation are linked to suicidal behaviors.

With the progression of adolescent substance use involvement, abnormalities in serotonin regulation may be related to differential expression of the serotonin transporter and low levels of brain serotonin, as well as other neurobiological processes (Schepis et al. 2008).

### Introduction to risk mechanisms

There are likely to be multiple mechanisms that determine how, when, and to what extent the above risk factors are related to suicidal behavior and substance use. Above, we introduced the concept that the combination of poor impulse control, stressful life events, and substance use may interact to amplify the likelihood of negative consequences of these behaviors (Bridge et al. 2006; Dalton et al. 2005; Putnins, 1995). In the next section, we present a detailed model of how these interactions may actually occur.

## Mechanisms Underlying Adolescent Suicidal Behavior and Substance Use

Suicide results from the confluence of stressors and other risk factors interacting with underlying predispositions or vulnerabilities (i.e. diatheses). Theoretical interrelationships between stressors, risk factors, and diatheses have been outlined by Mann and colleagues in their “stress-diathesis” model of suicide (1999) and extended in more recent work (Mann, 2003). In the stress-diathesis model, impulsivity is the proximal factor leading to suicidal behaviors, but other factors can modify the level of impulsivity to increase the risk for suicidal behaviors (Brent and Mann, 2003; Mann, 2003; Mann et al. 1999). In Mann’s original model of suicidal behavior, poor impulse control mediates the relationship between suicidal planning and suicide attempt. Other factors can modify the level of impulse control and increase the risk for suicidal behaviors (Mann, 2003; Mann et al. 1999). Impulsivity can be modified primarily through: (a) psychiatric state and life events; or (b) serotonin function and substance use. One pathway (**A** in the figure, below) is associated with psychosocial crises and life events that can lead to depression, hopelessness, and suicidal ideation, thereby worsening impulse control. The other pathway (**B**) is associated with serotonin dysregulation (which can be influenced by substance use), that can also worsen impulse control. These pathways can operate independently or concurrently. Suicidal behaviors generally occur through two types of diatheses or vulnerabilities: 1) major psychopathology (most commonly depression); and 2) impulsive-aggression and its neurobiological correlates (impaired executive function, and serotonin dysregulation in the ventral prefrontal cortex) (Brent and Mann, 2003; Mann et al. 1999; Shaffer and Pfeffer, 2001).

**Figure 2:**
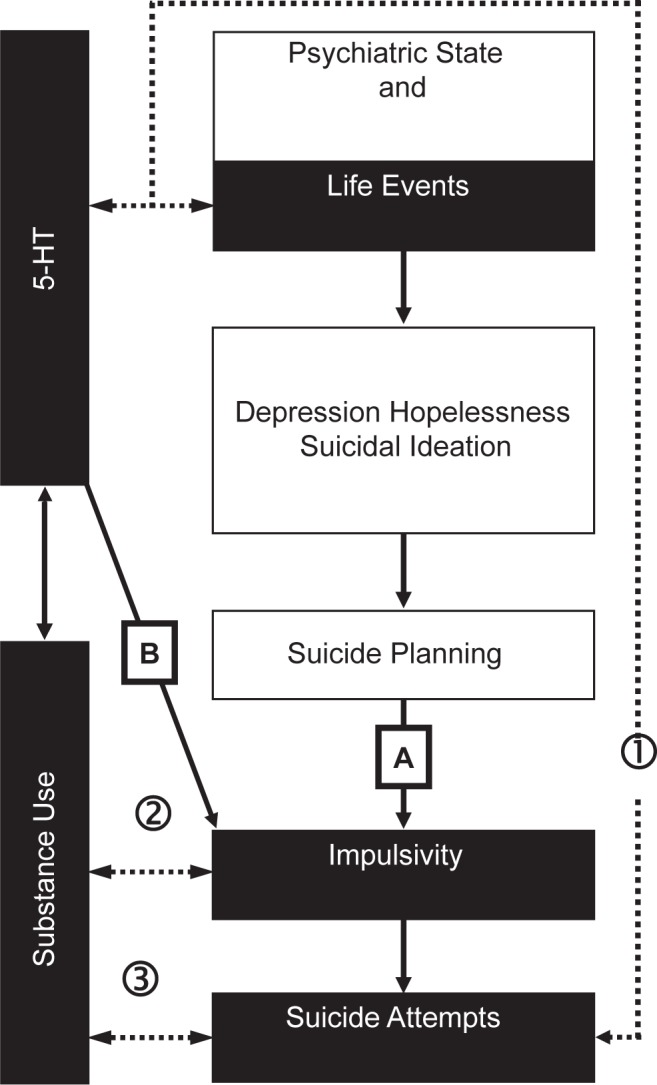
A development model of adolescent suicidal behavior and substance use (adapted from Mann et al. 1999).

To adapt the “stress-diathesis” model to explain adolescent suicidal behaviors and substance use, we propose that a developmental-transactional model is required that includes not only precursors and salient risk factors (Bridge et al. 2006; Windle, 2004), but also plausible mechanisms that explain how, when, and to what extent interactions among these risk factors occurs. Precursors include familial factors, such as parental mood disorders and parental impulsive aggression; exposure to these familial factors can lead to neuroticism, hopelessness, and mood symptoms in pre-pubertal offspring, and depression later in adolescence. Other precursors include parental impulsive-aggressive traits and parental suicide attempts, producing altered serotonin function, and deficits in executive functioning, leading to impulsive-aggressive traits in pre-pubertal and pubertal offspring. At least three causal pathways are hypothesized to underlie the vulnerabilities that lead to adolescent suicidal behaviors. These pathways (identified by ①, ②, and ③ in Fig. 2) address the possibilities of bidirectional interactions between (1) stressful life events, genetic markers of serotonin dysregulation, and suicide attempts; (2) substance use and impulsivity; and (3) substance use and suicide attempts.

From a developmental-transactional perspective, psychopathology (especially mood disorders) developing after the onset of puberty may lead to suicidal ideation (Bridge et al. 2006). Suicidal ideation may interact with stressful life events (such as legal problems, interpersonal loss, interpersonal discord), to produce a suicide attempt. Additional factors, such as current drug intoxication, exposure to suicide, or availability of a lethal agent, can further increase the risk of completed suicide (Bridge et al. 2006). More specifically, the developmental-transactional model provides a framework for research questions, such as: (a) does a genetic predisposition for 5-HT dysregulation interact with stressful life events, leading to elevated risk for suicidal behavior; (b) can the presence of suicidal or impulsive behaviors lead to substance use; and, conversely (c) can substance use increase levels of impulsivity or suicidal behaviors?

In regards to the question of whether a genetic predisposition for serotonin dysregulation interacts with stressful life events, we hypothesize that a genetic predisposition to serotonin dysregulation interacts with stressful life events in vulnerable individuals. For example, individuals with major depression (Kendler et al. 2005) or anxiety-related temperament (Pezawas et al. 2005) are thought to have reduced serotonin function at the level of the serotonin transporter (the transporter protein that most directly regulates serotonin function in the brain), making these individuals more sensitive to the effects of stressful life events (Anguelova et al. 2003; Kendler et al. 2005; Lotrich and Pollock, 2004). Recent findings in adolescents and young adults have shown that functional serotonin transporter polymorphisms interact with stressful life events to predict depression and suicidality (Caspi et al. 2003; Kendler et al. 2005; Eley et al. 2004), and substance abuse (Covault et al. 2007; Kaufman et al. 2004, 2006, 2007; Olsson et al. 2005).

With regards to questions such as: Can the presence of suicidal or impulsive behaviors lead to or worsen substance use; and, can substance use lead to increased levels of impulsivity or suicidal behaviors, we recognize that interactions in developmental-transactional models are often bidirectional (Bridge et al. 2006) and not orthogonal (Bridge et al. 2006; Dahl, 2004; Pratt, 2002; Steinberg, 2005). Therefore, both of these outcomes are theoretically possible. For example, suicidal or impulsive behaviors in early adolescence may increase the likelihood of developing substance abuse and related psychiatric comorbidity in later adolescence. In contrast, early substance use may exacerbate impulsive-aggression in early adolescence, leading to suicidal behavior later in adolescence.

Regarding potential underlying mechanisms that may explain how, when, and to what extent risk and protective factors interact, to increase or decrease the likelihood of suicidal and substance use behavior, it is clear that multiple factors are involved in a developmental-transactional framework. One possibility is that stressful life events trigger suicidal or substance use behaviors in an attempt to relieve or reduce stress (Goldston, 2004; Goldston et al. 2008). For instance, some adolescents report that they attempt suicide or engage in self-harm in an effort “to get relief ” or “to escape” (Boergers et al. 1998; Hawton et al. 1982; Kienhorst et al. 1995) or to “stop bad feelings” (Nixon et al. 2002; Nock and Prinstein, 2004). Furthermore, the relationship between suicidal behavior and substance use may result from a desire to escape problems or to “self-medicate” (Hufford, 2001; Khantzain, 1997). This “relief/escape” mechanism may result in the co-occurrence of suicidal and substance use behaviors. For example, suicidal ideation is associated with high levels of stress, and the anxiolytic or euphoric effect of particular substances may reduce stress (Light et al. 2003) or increase urge or desire to use drugs (Robinson and Berridge, 2000; Koob and Le Moal, 2001; Schepis et al. 2008). Furthermore, the progression of adolescent substance abuse may act as an amplifying feedback loop, where the development of a substance use disorder results in reciprocal impairments in neurobehavioral constructs, such as impulse control, and neurobiological processes. When specific external stresses occur, the combined effect may be heightened risk of suicidal behavior and ongoing substance abuse.

## Approaches to the Study of Adolescent Suicidal Behavior and Substance Use

In this section, we present approaches and concepts that are important to the understanding of how the above-described risk factors may interact to produce adolescent suicidal behavior and substance use.

### Translational approaches

Broadly stated, “translational research is the process of applying ideas, insights, and discoveries generated through basic scientific inquiry to the treatment or prevention of human disease” (NINDS PAR-05–158, September 6, 2005). Translational behavioral research requires flow of information from clinical settings to the laboratory and from the laboratory to the clinic. This bidirectional approach requires new pathways for discovery, including multi-disciplinary research teams that have expertise in the diverse areas being studied, such as adolescent suicidal behavior and substance use. To study adolescent suicidal and substance use behaviors from this perspective, collaborative research teams will need expertise in molecular and behavioral genetics, psychology, sociology, cognitive and affective neuroscience, developmental psychopathology, psychophysiology, brain imaging, and laboratory measures of behavior. The advantage of this translational approach as it applies to adolescent suicidal and substance use behaviors is that it will produce greater understanding of the risk mechanisms leading to these behaviors, which can ultimately inform treatment plans and preventive interventions. In the following sections, we focus on specific issues related to conducting translational studies of adolescent suicidal behavior and substance use.

### Multidimensionality of suicidal and substance use behavior

Suicide attempters and substance users form heterogeneous populations (e.g. Epstein et al. 2002; Goldston et al. 1998; Pfeffer et al. 1983; Walrath et al. 2001) with different risk and protective factors existing within subgroups of suicidal or drug-using individuals (Epstein et al. 2002; Goldston et al. 1996, 1998; Mandell et al. 2006; Walrath et al. 2001). Previous efforts to distinguish or characterize groups have generally focused on suicidal behavior or substance use behavior at a single time point, such as time-of-treatment. However, an individual’s substance use or suicidal behavior may change over time. Research is needed to characterize different developmental trajectories of suicidal and substance use and the risk and protective factors associated with these different trajectories.

### Dimensional versus categorical approaches

Debate continues between quantitative versus qualitative conceptualization of psychopathology in general and suicidal behavior or drug abuse in particular. Are suicidal and substance abuse behaviors dimensional and continuously distributed, with quantitative (but not qualitative) differences based on levels of severity or between continua of severity, with “pathological” and “non-pathological” existing at opposite ends of the distribution but without clear cut points? In contrast, are there actual cut points that demarcate discrete taxa or diagnostic subgroups (Hill, 2002; Hinshaw et al. 2002; Leboyer et al. 2005)? Arguably, psychopathology has properties that are amenable to dimensional and categorical assessment, and therefore both types of assessment should be used. Recent findings show that a dimensional analysis of suicidal behavior and related risk factors yield dimensions that are opposite and independent, such as under- versus over-engagement and rejection-turmoil (Hyde et al. 2005). Furthermore, it may be the case that suicidal behavior is itself an independent clinical outcome with both dimensional and categorical properties (Leboyer et al. 2005; Windle, 2004).

While formal diagnostic entities exist in the Diagnostic and Statistical Manual of Mental Disorders, the evidence is mixed regarding how these and other diagnostic categories, obtained from psychiatric interviews, relate to suicidal behavior and risk factors for suicide (see DSM-IV-TR, APA, 2000; Leboyer et al. 2005). For example, DSM criteria for a psychiatric disorder such as Major Depressive Episode states that a specific number of symptoms (5 or more of 9 symptoms) must be present during the same 2-week period and represent a change from previous functioning; at least one of the symptoms must be either depressed mood or loss of pleasure or interest. Suicidal behavior can include “recurrent thoughts of death (not just fear of dying), recurrent suicidal ideation without a specific plan, or a suicide attempt or a specific plan for committing suicide (DSM-IV-TR, APA, p. 356).” However, this categorical approach does little to quantify depressive and suicidal symptoms on a continuum that is related to risk for suicidal behavior. To assess dimensional aspects of depression and suicidal behavior, specific validated self-report measures should be used. Therefore, since adolescent suicidal behavior appears to have both categorical and dimensional properties, we recommend that both properties be studied, using psychiatric interviews and self-report measures.

Similar to the duality in conceptualization of suicidal behavior, there are two different methods for examining different aspects of substance use that include assessing sequential stages of use between different drug classes, or assessing patterns of use within a specific drug class. The onset of first use of different substances has often been characterized as occurring in sequential categorical stages (Kandel, 1975; Labouvie and White, 2002), which correspond to stages of escalation through different classes of drugs. These categorical stages begin with the first use of beer and/or wine, and then progress through first uses of hard liquor and/or cigarettes, marijuana, and finally use of multiple illicit drugs. This approach is used to describe the initiation of substance use involvement as a progression through drug classes. However, adequate classification of adolescent substance use patterns also requires consideration of how substance use progresses within each drug class. From this latter perspective, substance use progression within a drug class is viewed as transitions from initiation, continuation, and maintenance/escalation of use, to cycles of regression, cessation, and relapse (Clayton, 1992). Furthermore, progression within a class, using dimensional measures such as quantity and frequency of substance use, may predict progression to other classes of substance use and patterns of poly-substance use (Clayton, 1992; Clark et al. 2005). Similar to suicidal behavior assessments, we recommend assessments of both sequential (categorical) and progressive (dimensional) use patterns for adolescent substance use involvement.

### Multilevel developmental assessment approach

Developmental assessments of suicidal and substance use behaviors require the use of integrated multilevel bio-psycho-social models (Cicchetti, 1993; Hinshaw, 2002; Windle, 2004; Windle and Davies, 1999; Zucker et al. 1995). This method emphasizes that developmental outcomes are interdependent and influenced by multiple factors, such as genetic, biochemical, physiological, cognitive, and environmental variables. Using this method, assessments of the influences of these different types of variables are conducted concurrently to better understand suicidal and substance use behaviors. This developmental approach includes the principles of reciprocal causation such that an adolescent’s behavioral characteristics elicit particular behavioral responses from parents and peers, and these parents and peers influence the adolescent’s behavior, and interactions between the individual and their environment (e.g. gene-environment interactions and correlations). The goal of this approach is to examine patterns of interpersonal interactions and the behavioral outcomes that occur from these interactions across development (e.g. Caspi and Moffit, 1995; Caspi et al. 2003; Hinshaw, 2002; Sameroff, 2000). Testing these models requires a prospective design.

### Longitudinal designs and statistics

To identify when and how risk (and protective) factors interact to influence the likelihood of suicidal behaviors and substance use, longitudinal studies are needed to begin to understand causal relationships. Well-designed epidemiological and longitudinal studies will help to reduce bias that can complicate cross-sectional study from highly selected or clinical samples (Wilcox and Anthony, 2004). Prospective studies of samples recruited from clinical settings have the advantage of examining the predictors and correlates of suicidal behavior found in treatment populations of substance abusers (Wines et al. 2004) and previously hospitalized adolescents (Goldston et al. 2006). Prospective longitudinal designs also allow for study of developmental timing, duration, and severity of risk factors, as well as examination of etiologic mechanisms. For example, genetically informative prospective designs, such as children-of-twins studies, can be used to examine gene-environment interactions and correlations (Glowinski et al. 2004). Furthermore, case-crossover designs can be used to test hypotheses linking triggering mechanisms to recent substance use and suicidal behavior (Wilcox et al. 2004; Wu and Anthony, 2000).

By using random effects and latent class models, it should be possible to examine how suicidal and substance use behaviors interrelate with biological risk factors (e.g. 5-HT dysregulation), behavior (e.g. impulsivity and impulsive aggression), and environmental factors (e.g. stressful life events). *Latent class models* have been suggested as alternative methods for identifying subtypes of patients or substance users based on individual response patterns (e.g. Graham et al. 1991; Uebersax, 1994). Latent growth curve modeling is a statistical method for understanding growth patterns over time. More recently, an approach has been developed that combines latent class analysis and latent growth modeling (Muthén, 2001). Under this model, latent trajectory classes are derived that represent subgroups with similar growth trajectories or developmental patterns. The combined approaches are advantageous, since they allow classes to be defined based on their patterns of responses over time (Muthén, 2001). For example, these techniques were used to identify different patterns of aggressive responses to the “Good Behavior Game” prevention program for reducing aggression in the Baltimore city schools (Muthén, 2001). Such methods could be applied to identify groups with differing trajectories of suicidal and substance use behaviors, and have been suggested by the Institute of Medicine as an alternative method for analyzing suicidal behaviors (Goldsmith et al. 2002).

In closing this section, we next present approaches and concepts that are important to the understanding of how impulse control should be assessed in prospective longitudinal studies of adolescent suicidal behavior and substance use.

### Laboratory behavioral assessment of impulse control

The use of multiple approaches to studying impulse control is important because previous research and theory has conceptualized impulsivity as a multidimensional construct that requires multiple modes of measurement for accurate assessment (Barratt and Patton, 1983; Dougherty et al. 2003a, 2003g; Nigg, 2000). Using the operational definition of poor impulse control (Moeller et al. 2001; p. 1784) provides a broad conceptual framework from which multiple aspects of impulse control can be studied. There are three commonly used behavioral paradigms for assessing impulse control: continuous performance tests (CPT), stop tasks, and delay-discounting tasks (Dougherty et al. 2003a, 2003g). Impulsive responses yielded from each of these tasks are conceptualized as distinct processes in behavioral theories.

There are both theoretical and neuroanatomical differences that support the distinction between the measures of impulse control. Gray (1982, 1987) and Logan (1994) have each proposed models of behavior that contrast mechanisms of behavioral activation and inhibition that may be measured with laboratory behavioral tasks. Impulsive errors made during CPT tasks are the result of failures to process stimuli completely prior to responding (Dougherty et al. 1999b; Halperin et al. 1988, 1991), which could be attributed to problems with behavioral activation. In contrast, impulsive errors made during stop tasks are failures to inhibit an already initiated response, which could be attributed to problems with behavioral inhibition (Dougherty et al. 2005a). On the other hand, an inter-temporal choice mathematical model has been proposed by neuropsychiatric and behavioral researchers (Ohmura et al. 2005; Reynolds et al. 2004; Takahashi, 2004) to explain responses on delay-discounting tasks. This model corresponds to impulsive choices made during delay-discounting tasks, defined by choosing smaller-sooner reinforcers over larger-later reinforcers. A comparative factor analysis has demonstrated that each task assesses variance unique to that particular task (Dougherty et al. 2003a, p. 1153). In addition to these theoretical distinctions, there are also unique neuroanatomical processes that further support a distinction between tasks. When performing CPT tasks, brain activation occurs in the inferior and medial prefrontal cortex, and the inferior parietal cortex (Garavan et al. 1999). When performing stop tasks, brain activation occurs in the right medial, mesial, and inferior frontal cortex, and the left caudate nucleus (Aron et al. 2003; Casey et al. 2002; Rubia et al. 2001). For delay-discounting tasks, Bechara (2005) has proposed that a dynamic interaction exists between the amygdala and the ventral medial prefrontal cortex (including Brodmann’s areas in the medial orbitofrontal cortex). This interaction is supported by imaging studies, which have shown that brain activation during the performance of this task occurs in the orbitofrontal cortex, along with activation of the striatum (Rogers et al. 2004).

Besides the theoretical and neuroanatomical distinctions described above, cross-sectional studies have related performance on these three types of behavioral paradigms to suicidal and substance use behavior. Studies using CPTs have shown elevated impulsive responding among suicide attempters (Dougherty et al. 2004b, 2004c; Horesh, 2001; Mathias et al. 2001) and drug abusers (Moeller et al. 2002a, 2002b, 2004, 2005; Swann et al. 2004). Studies using stop tasks have shown that impulsive responding is elevated among suicide ideators (Mathias et al. 2001) and drug abusers (Fillmore and Rush, 2002; Kamarajan et al. 2005; Kaufman et al. 2003; McDonald et al. 2003; Moeller et al. 2002b). Studies using delay-discounting tasks have shown elevated impulsive choices among suicide ideators (Mathias et al. 2001), suicide attempters (Mathias et al. 2006), and drug abusers (Allen et al. 1998; Coffey et al. 2003; Kirby et al. 1999; Odum et al. 2000; Petry, 2002). Finally, while different types of suicidal and drug-abusing populations have exhibited poor impulse control, studies have typically relied on a single type of behavioral measure administered at a single time point. Assessments of multiple components of impulse control, both as predictors and ongoing behaviors across time are needed to determine how different characteristics of impulsivity relate to suicidal and drug-use behaviors (Dougherty et al. 2004b; Erinoff et al. 2004).

## Summary and Future Directions

As the suicidal behavior-substance abuse relationship is particularly common in adolescence, and is increasingly the focus of applied and clinical research, there are important design and conceptual issues that should be considered. Prospective longitudinal studies should include samples from both epidemiologically representative community samples as well as clinical samples. Innovative prospective designs and statistics using random effects and latent class models are recommended. Future prospective studies should aim to understand causal relationships. To the extent that these issues are addressed, progress will be made in understanding how and when suicidal behavior-substance use relationships occur in adolescence. Knowledge gained from these prospective longitudinal studies should lead to greater understanding on the timing, duration, and extent to which specific risk and protective factors influence the outcomes of adolescent suicidal behavior and substance abuse. In turn, findings from these studies should inform researchers who conduct future treatment and prevention studies.
